# Perspectives in membranous nephropathy

**DOI:** 10.1007/s00441-021-03429-4

**Published:** 2021-04-06

**Authors:** Nicola M. Tomas, Tobias B. Huber, Elion Hoxha

**Affiliations:** grid.13648.38 0000 0001 2180 3484 III. Department of Medicine, University Medical Center Hamburg-Eppendorf, Hamburg, Germany

**Keywords:** Membranous nephropathy, PLA2R, THSD7A

## Abstract

The identification of the phospholipase A2 receptor 1 (PLA2R) and thrombospondin type-1 domain-containing protein 7A (THSD7A) as podocyte antigens in adult patients with membranous nephropathy (MN) has strongly impacted both experimental and clinical research on this disease. Evidence has been furnished that podocyte-directed autoantibodies can cause MN, and novel PLA2R- and THSD7A-specific animal models have been developed. Today, measurement of serum autoantibody levels and staining of kidney biopsies for the target antigens guides MN diagnosis and treatment worldwide. Additionally, anti-PLA2R antibodies have been proven to be valuable prognostic biomarkers in MN. Despite these impressive advances, a variety of questions regarding the disease pathomechanisms, clinical use of antibody measurement, and future treatments remain unanswered. In this review, we will outline recent advances made in the field of MN and discuss open questions and perspectives with a focus on novel antigen identification, mechanisms of podocyte injury, clinical use of antibody measurement to guide diagnosis and treatment, and the potential of innovative, pathogenesis-based treatment strategies.

## Introduction

**Fig. 1:**
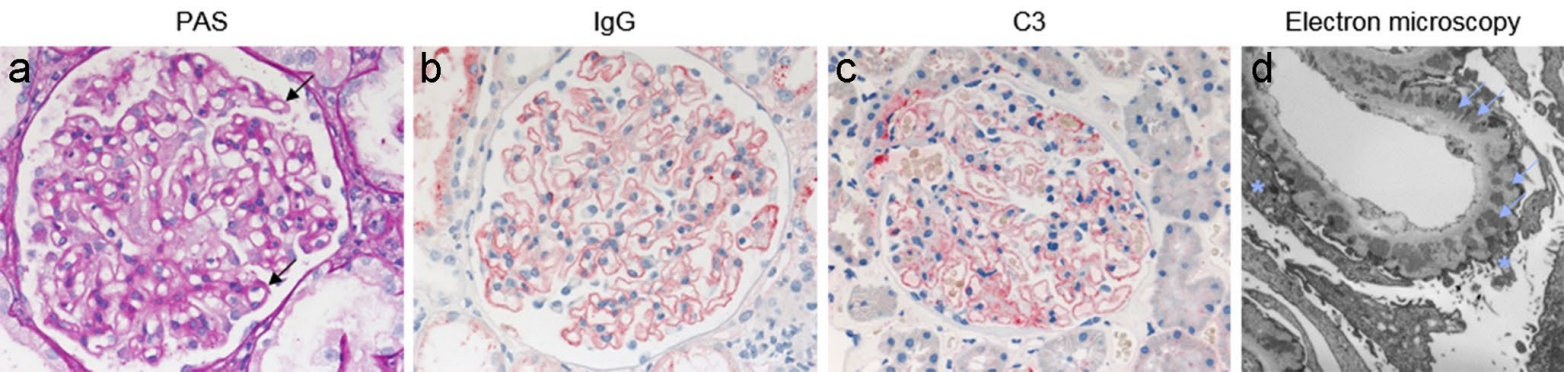
Morphological findings in a patient with membranous nephropathy. (**a**) Periodic acid Schiff (PAS) staining demonstrates prominent glomerular capillary walls (black arrows) as a sign of a thickened glomerular basement membrane (GBM). (**b**, **c**) Immunohistochemical staining for IgG (**b**) and complement component C3 (**c**) shows strong positivity (red), indicating deposition of (auto)antibodies and C3. (**d**) Electron microscopy demonstrates a thickened GBM with electron dense deposits accentuated at the outer (subepithelial) aspect of the GBM (light blue arrows) and effacement of podocyte foot processes (light blue asterisks). Images kindly provided by Thorsten Wiech, University Medical Center Hamburg-Eppendorf

Membranous nephropathy (MN) represents the most common cause of a nephrotic syndrome in Caucasian adults, accounting for 20–30% of histopathological diagnoses in patients with a urinary protein excretion (i.e., proteinuria) of more than 3.5 g per day (Haas et al. 1997; Braden et al. 2000). MN is histomorphologically defined by a diffuse thickening of the glomerular capillary wall in light microscopy, which is particularly evident in the later stages of the disease. Immunohistochemical stainings reveal a granular positivity for immunoglobulins and complement components, suggesting a role of these molecules in the pathogenesis of MN. The hallmark findings in electron microscopy include electron-dense deposits in a strictly subepithelial localization, i.e., at the outer aspect of the glomerular basement membrane (GBM), and an extensive effacement of podocyte foot processes. Ehrenreich and Churg proposed a histomorphological classification with stages I to IV based on electron microscopy (EM) findings such as immune deposits and GBM thickness (Ehrenreich T et al. 1968). The typical histomorphological signs of MN are exemplified in Fig. 1.

Historically, MN was classified either as primary or secondary MN (Glassock 2010). Secondary MN occurs in association with other diseases such as systemic lupus erythematosus (SLE), infections and malignancies, or exposure to toxins as wells as certain medications such as non-steroidal anti-inflammatory drugs, penicillamine, probenecid, and TNF alpha blockers. Primary MN is considered to be an antibody-mediated autoimmune disease; therefore, different immunosuppressive treatment protocols were developed, usually consisting of alkylating agents combined with steroids, calcineurin inhibitors with or without steroids, or the B cell-depleting agent rituximab (Ponticelli et al. 1998, Cattran et al. 1999, Cattran et al. 2001, Howman et al. 2013, Ruggenenti et al. 2015, Dahan et al. 2017, van den Brand et al. 2017, Fervenza et al. 2019). Because of the different treatment approaches, discriminating primary from secondary MN is very important. Besides the clinical distinction on the basis of the presence or absence of an associated disease, pathological findings are also used to differentiate primary from secondary MN. In primary MN, IgG deposits usually show a predominance of IgG4, while in some secondary MN entities, e.g., SLE, mainly non-IgG4 subclasses are detected and IgA or IgM may also be present (Ohtani et al. 2004; Segawa et al. 2010). A non-IgG4 predominance in the kidney biopsy has also been described for patients with malignancy-associated MN, but findings on glomerular IgG subclass deposition in patients with primary or tumor-associated MN are inconsistent throughout the literature (Murtas et al. 2016; Hoxha et al. 2017; von Haxthausen et al. 2018). Additionally, it is difficult to differentiate a causal link between the diagnosis of a tumor and the development of MN from a mere coincidence, in particular as MN patients are often older and have a higher risk for a tumor.

Another challenge for the management of patients with MN is the fact that one third of patients develop a spontaneous remission of disease with an excellent outcome, while another 20–30% of patients develop chronic kidney disease with an eventual need of renal replacement therapy. Considering the potentially severe toxicity of the available immunosuppressive treatments, biomarkers are needed to accurately predict the natural history and prognosis of disease. The development of such biomarkers requires a better understanding of the disease pathogenesis.

The most extensively used animal model to study the pathophysiology of MN is the rat model of Heymann nephritis (Heymann 1952; Heymann et al. 1959). The passive Heymann nephritis model is induced by transfer of heterologous antibodies from sheep or chicken, which were immunized with rat brush border protein extracts. Subsequently, rats develop antibodies against the foreign IgG, dividing the model into a heterologous phase (when disease is mediated by the heterologous antibodies, lasting for five to seven days after antibody injection) and an autologous phase (when disease is exacerbated by the developing antibodies) (Kerjaschki et al. 1996). The active model is induced by immunization of rats with rat brush border protein extracts in combination with Freund’s adjuvant. These animal models were key for our understanding of MN pathophysiology over the last decades, including the following: (i) immune deposits develop in situ after binding of circulating (auto)antibodies to an endogenous podocyte antigen; (ii) antibody binding and deposition along the glomerular filtration barrier (GFB) activate the complement system, which may contribute to the glomerular damage in MN. The key antibodies in the Heymann nephritis models were later identified to be directed against megalin (gp330) (Kerjaschki et al. 1982), which, however, is not expressed on human podocytes and therefore cannot operate as a disease-mediating agent in patients with MN. Consequently, a long-winded search for the human autoantigen(s) in MN was initiated and substantial progress was made over the past decade.

## Antigens in patients with MN

### NEP

In 2002, neutral endopeptidase (NEP) was identified as the first autoantigen in neonate patients with MN (Debiec et al. 2002). Neonates developed MN due to vertical transfer of anti-NEP antibodies from a genetically *NEP*-deficient mother that was pre-immunized with NEP, e.g., in the context of a previous miscarriage or pregnancy (Debiec et al. 2002, 2004; Vivarelli et al. 2015). Purified IgG fractions from the mothers’ sera were transferred to NEP-expressing rabbits, which resulted in subepithelial immune complexes and proteinuria, confirming the in situ nature of immune complex formation. However, anti-NEP antibodies are not found in adults with MN and thus NEP-associated MN represents only a rare entity with a few described cases worldwide (Ronco et al. 2020).

### PLA2R

**Fig. 2:**
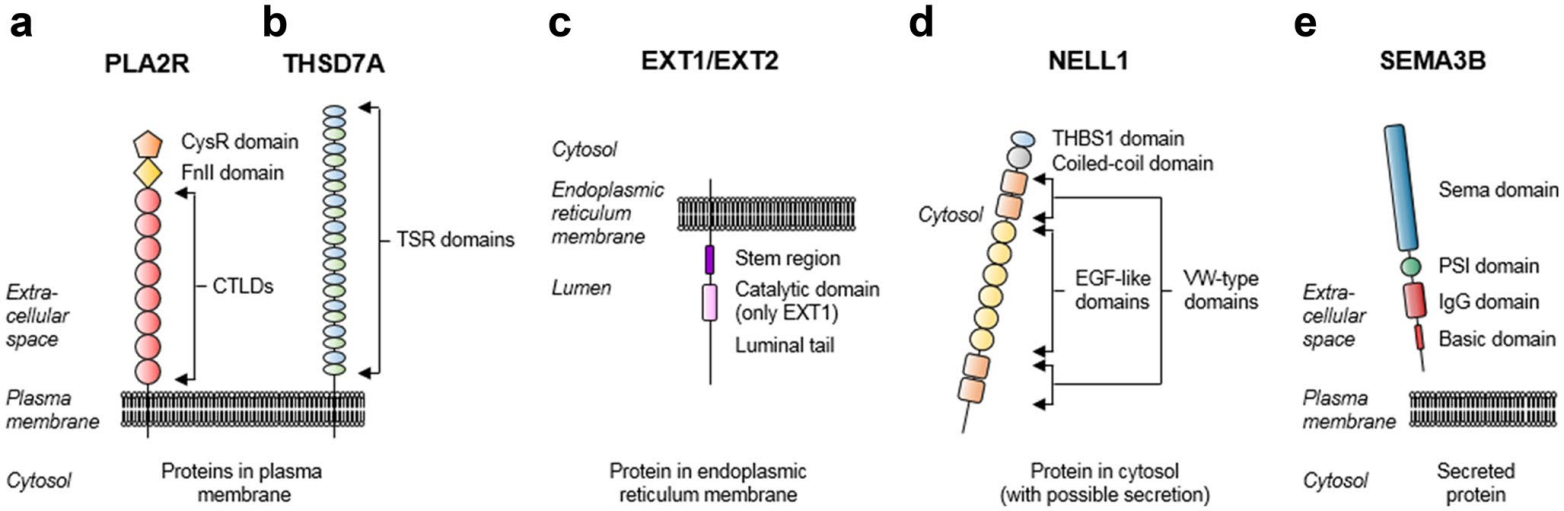
Molecular architecture of MN antigens/biomarkers. (**a**) PLA2R is a 180 kDa plasma membrane protein consisting of a cysteine-rich (CysR) domain, a fibronectin type II (FnII) domain, eight c-type lectin domains (CTLDs), one transmembrane region and a short cytoplasmic tail. (**b**) THSD7A is a 250 kDa plasma membrane protein consisting of twenty-one thrombospondin repeat (TSR) domains, which can be further classified as thrombospondin (THBS1)-like (blue) or F-spondin/C6-like (green), one transmembrane region and a short cytoplasmic tail. (**c**) EXT1 and EXT2 are proteins expressed in the endoplasmic reticulum membrane with a short cytoplasmic tail, one transmembrane region, short stem region and a long luminal tail. EXT1 contains a luminal catalytic domain. (**d**) NELL1 is a 90 kDa cytosolic protein containing a THBS1-like domain, one coiled-coil domain, 4 von Willebrandt (VW)-type domains and six epidermal growth factor-like (EGF-like) domains. (**e**) SEMA3B is a 80 kDa secreted protein containing a Sema domain, a plexin-semaphorinintegrin (PSI) domain, an immunoglobulin (IgG) domain and a basic domain

It was not until 2009 when phospholipase A2 receptor 1 (PLA2R) was discovered as the first autoantigen in adult patients with primary MN (Beck et al. 2009). In their landmark study, the authors tested patient sera on extracts of human glomeruli (derived from apparently healthy kidneys) using Western blotting. Thereby, their experimental approach was based on the fact that antigen recognition by many autoantibodies depends on the three-dimensional conformation of the antigen. To better preserve the structure of the putative antigen(s) in the glomerular extracts, the authors removed the reducing agent from the experimental conditions, which resulted in the recognition of a high molecular weight and heavily glycosylated protein by the circulating autoantibodies. Using partial purification and mass spectrometry, this protein could be identified as PLA2R. Systematic testing of further patient sera revealed that around 70% of MN patients have circulating anti-PLA2R antibodies, identifying PLA2R as the major autoantigen in patients with MN (Beck et al. 2009). PLA2R is a member of the mannose receptor family and consists of one cysteine-rich domain, one fibronectin type II domain, eight c-type lectin domains, one transmembrane region and a short intracellular tail (Lambeau et al. 1994; East et al. 2002) (Fig. 2a). Apart from podocytes in the kidney, PLA2R is also expressed in neutrophils, pulmonary macrophages, airway submucosal epithelium, and airway columnar epithelial cells (Silliman et al. 2002; Granata et al. 2005; Nolin et al. 2016). Moreover, in addition to the membrane-bound form of PLA2R that is expressed on cell surfaces, there is also a soluble form of PLA2R that can be found in serum of healthy individuals (Watanabe et al. 2018a, b).

### THSD7A

An international collaborative effort led to the identification of the second autoantigen in adult patients with MN, thrombospondin type-1 domain-containing protein 7A (THSD7A) (Tomas et al. 2014). Using a similar approach as for the identification of PLA2R, anti-THSD7A autoantibodies were detected in around 10% of anti-PLA2R antibody negative cases (thus representing around 3% of total MN cases). THSD7A consists of 21 extracellular domains, which are all thrombospondin type-1 repeat (TSR) domains (Seifert et al. 2018; Stoddard et al. 2019). These TSR domains can be further classified as thrombospondin (THBS1)-like or F-spondin-like, the latter sometimes also referred to as complement component C6-like (Fig. 2b).

Both PLA2R and THSD7A are single-pass transmembrane proteins of high molecular weight with multiple repetitive extracellular domains that are rich in disulfide bonds. The expression of PLA2R and THSD7A in the membrane of podocyte foot processes rather than in the cytosol or intracellular compartments ensures their accessibility and thus supports the assumption that circulating autoantibodies can induce disease after binding to these molecules in situ. Moreover, PLA2R- and THSD7A-specific antibodies are predominantly of the IgG4 subclass, the least abundant IgG subclass in healthy human serum (Beck et al. 2009; Tomas et al. 2014). Although rare cases of dual anti-PLA2R and anti-THSD7A antibody positivity have been described (Larsen et al. 2016; Zaghrini et al. 2019), these common features indicate that PLA2R and THSD7A are primary antigens in MN and that PLA2R- and THSD7A-associated MN represent distinct molecular entities.

Importantly, both PLA2R and THSD7A-associated MN can be diagnosed not only by detection of circulating antibodies, but also by biopsy staining for the PLA2R and THSD7A antigens, which usually reveals an increased signal in the respective cases (Debiec et al. 2011; Hoxha et al. 2012; Tomas et al. 2014). Notably, biopsy staining is more sensitive than serum antibody measurement, in particular if the diagnosis is made retrospectively and serum antibody levels have already decreased as a consequence of immunological remission (Hoxha et al. 2012, 2017; Svobodova et al. 2013).

### Other recently defined MN subgroups

PLA2R- and THSD7A-associated cases represent, depending on the patient cohort, 75 to 85% of total primary MN cases. This leaves 15 to 25% of patients with an unknown target antigen. In 2019, the proteins exostosin 1 and 2 (EXT1 and EXT2) were identified in mass spectrometric analyses of laser-dissected glomeruli from patient biopsies which were negative for PLA2R staining (Sethi et al. 2019). EXT1 and EXT2 are glycosyltransferases that are expressed in the endoplasmic reticulum membrane (Duncan et al. 2001) (Fig. 2c). The glomerular presence of EXT1 and EXT2 in patients with MN could be confirmed by immunohistochemical staining. Interestingly, most of the patients had clinical and laboratory findings of associated autoimmune diseases (e.g., systemic lupus erythematosus) and the biopsies showed features of secondary MN such as IgM deposition and IgG1 predominance. In these cases, no circulating antibodies against EXT1 and EXT2 were detected in the available patient sera, challenging the concept of EXT1 and EXT2 as target antigens in MN.

More recently, neural epidermal growth factor-like 1 protein (NELL1) was described to be deposited in glomeruli in adult patients with MN. Using essentially the identical methodology as for the identification of EXT1/EXT2, the authors identified, out of 210 PLA2R-negative cases, a total of 34 cases positive for NELL1 by mass spectrometry and/or immunohistochemistry. Serum was only available from 5 out of the 34 NELL1-positive cases and anti-NELL1 antibodies could be detected in these sera. Although both the specificity of NELL1 for MN and its presence independent from anti-PLA2R or anti-THSD7A antibodies could be demonstrated in this study, several points demand particular attention: first, glomerular IgG was predominantly of the IgG1 subtype, which is typically considered as an indicator of secondary MN and stands in contrast to PLA2R- and THSD7A-associated MN, where IgG4 is dominant. Second, no glomerular NELL1 could be detected in control biopsies (including time 0 kidney transplant biopsies) and NELL1-positivity in MN patients was localized more to the GBM than the podocyte. Third, NELL1 is not a membrane-expressed molecule but rather localizes to the cytoplasm or is secreted (Kuroda et al. 1999), limiting its accessibility for circulating antibodies at the GFB (Fig. 2d). Additionally, a recent study reported that 33% of NELL1-associated MN cases also had a tumor history, indicating a possible association between NELL1-associated MN and malignancies (Caza et al. 2020). Therefore, even though NELL1 positivity in the kidney biopsy might depict a distinct subgroup of patients with MN, several important questions remain unanswered: are anti-NELL1 antibodies causative of MN, even though the antigen does not seem to be endogenously expressed on podocytes? May these antibodies be generated in the context of a “second hit,” i.e., when local damage is already present and certain proteins are up-regulated and ectopically expressed at the cell membrane or secreted from the cell? Similar findings were described for other intracellular MN antigens such as aldose reductase, SOD2 and alpha enolase (Prunotto et al. 2010; Bruschi et al. 2011), and a recent publication suggested that measurement of these antibodies might have a predictive value (Ghiggeri et al. 2020). In these cases, which “first hits” lead to such local damage? At the same time, no circulating immune complexes containing NELL1 were reported in NELL1-associated MN. Further studies are needed to understand of the role of NELL1 in these MN cases. The most recently described MN antigen is semaphorin 3B (SEMA3B), which was identified using the same mass spectrometry-based approach as for EXT1/EXT2 and NELL1 (Sethi et al. 2020). SEMA3B is, similar to NELL1, not a membrane but a secreted protein (Alto et al. 2017) (Fig. 2e). Interestingly, semaphorin 3B-associated MN was particularly often diagnosed in pediatric patients.

### Perspectives in novel antigen identification

Despite these recent advances, there is still a relevant number of patients with undefined antigens and pathogenesis of disease. So far, two different strategies were applied for antigen identification. In the case of PLA2R and THSD7A, sera were probed on protein lysates of apparently healthy kidneys. This approach allowed to identify circulating antibodies, which bind to antigens that are endogenously expressed in healthy glomeruli. Subsequently, it was confirmed that both antigens accumulate in the immune deposits of diseased glomeruli. In the cases of EXT1/EXT2, NELL1, and semaphorin 3B, the antigens were detected in glomeruli microdissected from patient biopsies. This approach allowed detection of antigens that are present only under disease conditions, which may also be the case when the antigen is overexpressed locally in the context of a “second hit” or is deposited as a planted antigen or as part of a circulating immune complex. Although these strategies complement each other and may lead to the identification of further antigens, it seems possible that they will not unravel the whole repertoire of MN antigens. Several obstacles are encountered during the search for more autoantigens and thus make the application of novel methodological strategies necessary: (i) autoantibody binding to the antigen can depend on the native (i.e., fully folded) conformation of the antigen with the consequence that even mild alterations in antigen structure, e.g., by denaturation due to the presence of sodium dodecyl sulfate in standard Western blot conditions, prevent such binding. This can possibly be overcome by applying experimental techniques such as native blotting that preserve protein conformation. (ii) Intrinsic expression or extrinsic deposition of an antigen may be very low, limiting the ability to identify it by mass spectrometric analysis of standard samples. This could be circumvented by enrichment of the antigen, e.g., by purification of deposited IgG, which should be bound to the target antigen. However, such an approach would require the availability of larger amounts of frozen tissue, which is unfeasible in the clinical context. Another strategy to circumvent the issue of low antigen abundance in the human sample could be the probing of patient sera on protein arrays, identifying circulating antibodies against several thousands of defined peptides (Delville et al. 2014). However, the evident conformation-dependency of epitope recognition by patient autoantibodies may largely limit the use of this technique for the identification of novel antigens in MN. (iii) Groups of patients with recognition of the same still unknown antigen(s) may be very small or even restricted to single patients with the consequence of lacking samples for proper validation. Thus, the identification of relevant antigens on a “per-patient” level can be expected to be very challenging.

## Etiology and pathogenesis of MN

### Genetics

A genome-wide association study including 556 patients with MN in Europe identified two chromosomal loci that are associated with MN: the gene encoding HLA-DQA1 and, strikingly, the gene encoding PLA2R (Stanescu et al. 2011). In the presence of homozygosity for the risk alleles in the *HLA-DQA1* and *PLA2R1* genes, odds ratios for MN increased 20- and 4-fold, respectively. Interestingly, the odds ratio for MN increased to 80-fold in the homozygous presence of both risk alleles due to interaction between the two loci. The relevance of these two risk alleles was confirmed in several studies in European and Asian patients (Coenen et al. 2013; Lv et al. 2013; Ramachandran et al. 2016; Sekula et al. 2017). Moreover, *HLA-DRB1* has emerged as an additional risk allele in MN (Cui et al. 2017; Le et al. 2017) and studies have reported ancestry-specific effects of the classical HLA alleles: DRB1*1501 in East Asians, DQA1*0501 in Europeans, and DRB1*0301 in both ethnicities (Xie et al. 2020). The latter study, conducted in 3782 cases of East Asian and European ancestries, again confirmed risk alleles in the *PLA2R1* locus and discovered two novel loci encoding *NFKB1* and *IRF4*, which were associated with MN. This finding is particularly interesting since it suggests that the NK-κB signaling pathway and inflammatory processes play a role in the pathogenesis of MN. While the connection between genetic susceptibility due to carriage of certain risk alleles and the development of MN has been demonstrated in several studies and patient cohorts, the mechanistic basis of these associations, e.g., the phenotypic relevance of noncoding risk alleles in the *PLA2R1* gene and the effect of *HLA-D* variants on antigen presentation to T cells and the subsequent autoantibody response, need further clarification.

### Disease initiation

The molecular pathomechanisms of disease initiation and autoantibody formation in patients with MN have remained largely obscure. Generally, genetic predisposition (as described above), environmental factors, and a dysregulation of the immune system are considered to be the major triggers of autoimmune diseases. The identification of potential causes of loss of tolerance to certain antigens is made very challenging by the fact that autoantibodies can be present in the circulation for years or even decades before the disease is diagnosed, as shown in a recent study in PLA2R-associated MN (Burbelo et al. 2017). Therefore, the processes leading to loss of immune tolerance for PLA2R are not understood in the context of PLA2R-associated MN. Since these processes may take place years before MN becomes clinically apparent, and there are no active animal models of humanPLA2R-associated MN, their investigation is still a challenge. Thereby, autoreactivity to PLA2R can take place in a number of organs and tissues, which express PLA2R in the human body and is not constricted to the podocyte surface, which in normal conditions is to a certain extent an immunologic niche. In the case of secondary MN, several potential triggers such as malignancies and infections have been known for a long time. However, the underlying molecular basis of how and why autoantibodies are formed in these cases has been likewise unclear. In the last years, several mechanisms have been proposed that could explain the formation of autoantibodies and the subsequent development of MN.

PLA2R and THSD7A are not only expressed in the podocyte, but also several other tissues including the lung (von Haxthausen et al. 2018). A study from China found an increased frequency of MN in areas with higher air pollution (Xu et al. 2016). Moreover, a longer exposure to higher air pollution was associated with an increased risk to develop MN. This stands in contrast to areas with markedly lower air pollution such as Japan and Korea, where MN incidence is decreasing (Chang et al. 2009; Sugiyama et al. 2013). Studies in animal models revealed that intranasally instilled fine particles such as crystalline silica can induce adaptive immunity to generate autoantibodies (Brown et al. 2004). Obviously, these associative epidemiological data will need experimental studies to clarify whether such local inflammatory processes can lead to autoantibody formation and development of MN. It was shown that patients with MN have increased interleukin (IL)-4-positive cells and that the number of these cells correlates with proteinuria (Masutani et al. 2004). Two other studies identified low numbers of T regulatory cells (Tregs) in patients with MN (Roccatello et al. 2016; Rosenzwajg et al. 2017). Treg frequencies increased after treatment with rituximab, indicating restoration of immune balance. In line with these results, MN patients were recently identified to have higher Th17 and Th2 cytokines (IL-17A, IL-6, and IL-4) and lower Th1 and Treg cytokines (interferon-γ and IL-10), showing a cytokine dysbalance in MN patients (Cremoni et al. 2020). Interestingly, patients with the highest Th17 signature lived in areas with a higher burden of fine particulate matter, suggesting a connection between air pollution, immune dysregulation and incidence of MN. Collectively, these studies indicate that an immune dysregulation may play a relevant role in MN pathogenesis.

The incidence of different cancers is significantly higher in MN patients than in the general population (Lefaucheur et al. 2006). It was historically assumed that tumors can cause MN via the deposition of tumor antigens in the kidney with consecutive formation of autoantibodies or via the deposition of circulating immune complexes consisting of tumor antigens and antibodies. However, recent studies in patients diagnosed with THSD7A-associated MN and THSD7A-expressing tumors have challenged this concept (Hoxha et al. 2016). Malignancies can be found in around 20% of patients with THSD7A-associated MN (Hoxha et al. 2016, 2017) and were diagnosed within a median time of 3 months from the diagnosis of MN, strongly suggesting a causal relationship between the occurrence of the two diseases. Strikingly, in two patients, one with gallbladder carcinoma and one with endometrial carcinoma, strong expression of THSD7A was detected in the cells of the primary tumor and lymph node metastases. Moreover, THSD7A was also present in follicular dendritic cells of tumor-infiltrated lymph nodes. Considering the role of follicular dendritic cells for the production of antibodies by B cells (Heesters et al. 2014), a potential mechanism linking malignancy and MN was hypothesized, by which the immune system might have recognized THSD7A as a foreign antigen in the tumor cells, leading to production of high-affinity antibodies, which bind to THSD7A on podocytes in situ, forming subepithelial immune deposits, and initiating MN (Hoxha et al. 2016, 2017). In a subsequent systematic analysis of THSD7A expression in malignant tissues, the protein was found to be expressed in many neoplasias (Stahl et al. 2017). Together, these findings further support the concept of in situ antibody binding to podocytes, also in the case of tumor-associated MN, and on the other hand emphasize the complex interplay between a tumor and the immune system and how this connects to emergence of an autoimmune disease, challenging the classical concept of primary and secondary MN.

**Fig. 3:**
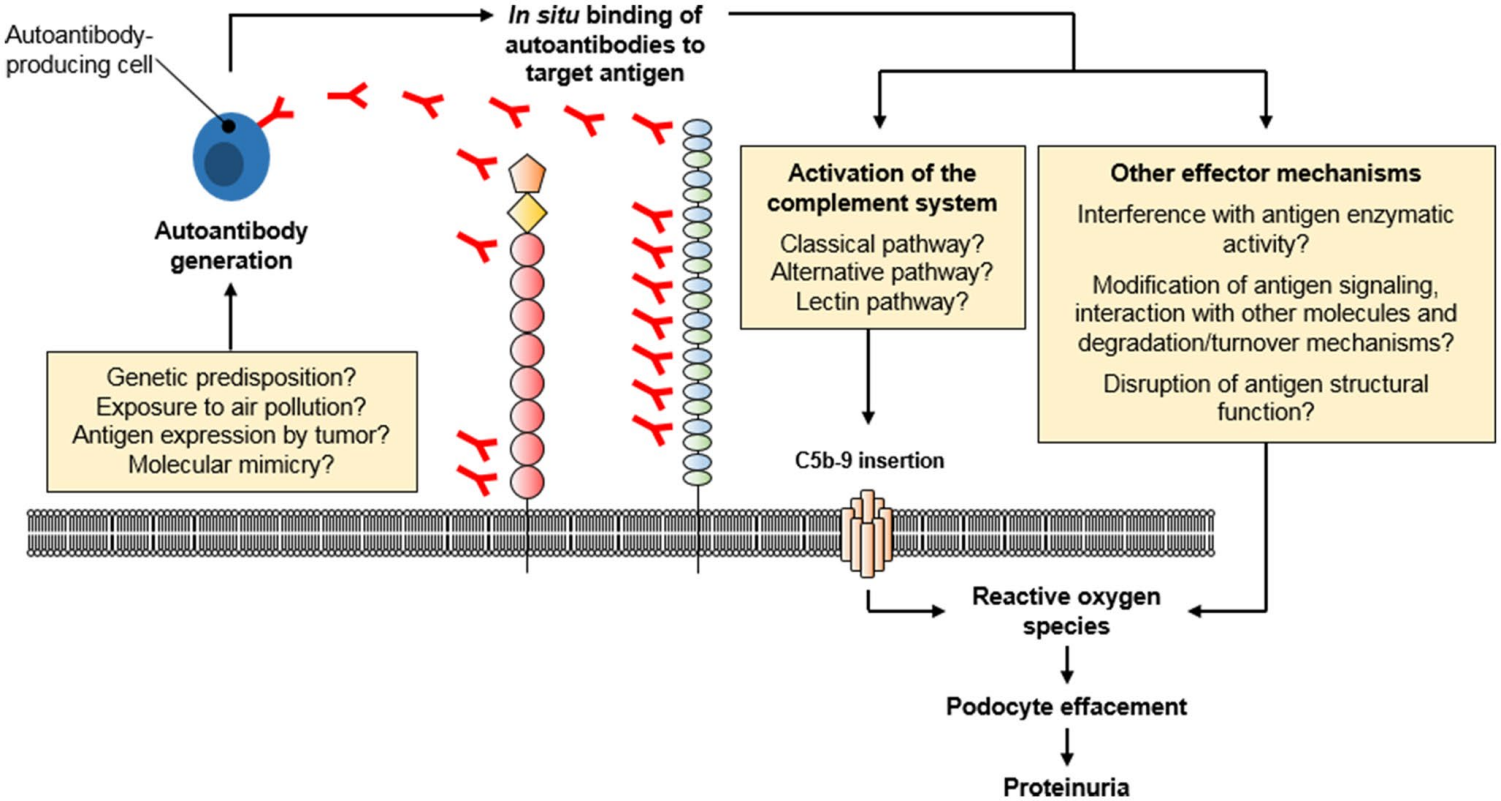
Molecular pathogenesis of MN. The initial step in the development of MN is the generation of autoantibodies. The initiation triggers of autoantibody generation have not been resolved, but several mechanisms (or a combination thereof) have been proposed: carriage of certain risk alleles, long-time exposure to air pollution, antigen expression by malignant tumors and molecular similarities of the antigens with, e.g., pathogens. Autoantibodies then bind to their target antigens on podocytes, which leads to the local activation of the complement system. Additional mechanisms of podocyte injury, such as disturbance of antigen function and signaling, have been suggested to play a role in MN pathogenesis, yet this needs further clarification

Amino acid sequence similarities between self and foreign proteins can lead to development of cross-reactivity antibodies, which after being initially generated against a pathogen, also bind endogenous proteins. Such foreign peptides may also activate autoreactive B and T cells (Rojas et al. 2018). This mechanism is usually referred to as molecular mimicry and has been suggested to be relevant in autoimmune diseases such as ANCA vasculitis, anti-glomerular basement membrane disease (Kain et al. 2008; Ooi et al. 2019; Gu et al. 2020), and myasthenia gravis (Schwimmbeck et al. 1989). Indeed, a 31-mer peptide in the cysteine-rich domain of PLA2R was identified that is recognized by sera from patients with PLA2R-associated MN and that shows sequence homology with a cell wall enzyme that is found in bacteria such as *Clostridium*, S*accharomyces cerevisiae*, and *Pseudomonas HrcC Type* (Fresquet et al. 2015), raising the possibility that exposure to microbes may play a role in the development of autoantibodies in MN*.* However, more clinical and experimental evidence is needed to support the hypothesis that molecular mimicry can lead to the formation of autoantibodies in this context. Taken together, the mechanisms of loss of tolerance to MN antigens with subsequent autoantibody formation (Fig. 3, left) are poorly understood and represent an exciting research area to follow in the next years.

### Animal models of MN involving THSD7A and PLA2R

The pathogenicity of human anti-podocyte antibodies in MN was for the first time demonstrated in NEP-associated MN (Debiec et al. 2002). In this study, isolated patient IgG induced subepithelial immune deposits and proteinuria in rabbits. In 2016, it was shown that anti-THSD7A antibodies isolated from the blood of patients with THSD7A-associated MN can induce the histological and clinical features of MN. Anti-THSD7A antibodies were transferred to wild-type mice, which express THSD7A on the podocytes. These studies demonstrated the pathogenicity of autoantibodies directed against an antigen that is also targeted in adult patients with MN (Tomas et al. 2016). Importantly, in this study, the THSD7A-specific antibodies were purified from patient sera using a THSD7A-coated column, thereby depleting the sera of the anti-THSD7A antibodies while leaving all other antibodies and serum components unchanged. Injection of sera depleted of anti-THSD7A antibodies in mice served as the control condition, thus excluding pathogenicity of serum components in the absence of anti-THSD7A antibodies. Interestingly, complement deposits were only found very late in the observation period (after 10 weeks). In experiments using a primary culture of mouse podocytes, anti-THSD7A antibodies induced cytoskeletal rearrangement with induction of focal adhesion signaling, hinting to complement-independent pathogenic mechanisms.

As THSD7A-associated MN is a rare entity, the establishment of an MN model independent from patient autoantibodies is important. To achieve this, anti-THSD7A antibodies were generated in rabbits and transferred to wild-type mice, which again induced the histological signs of MN and proteinuria (Tomas et al. 2017). Again, no deposition of complement was found, possibly due to reduced complement-activating capacity of the applied rabbit antibodies, and in vitro experiments demonstrated alterations in cell architecture after treatment with rabbit anti-THSD7A antibodies.

The studies using human anti-THSD7A antibodies in mice were feasible since THSD7A is expressed on mouse podocytes sharing 90% amino acid sequence homology with the human protein and patient autoantibodies efficiently bind mouse THSD7A (Godel et al. 2015; Meyer-Schwesinger et al. 2015; Tomas et al. 2016). By contrast, PLA2R is not expressed on mouse podocytes. Efforts to establish an animal model expressing the human PLA2R on the podocyte surface have so far been unsuccessful. However, a transgenic mouse line expressing murine PLA2R specifically in podocytes was recently developed (Meyer-Schwesinger et al. 2020). PLA2R expression was well tolerated and transfer of anti-PLA2R antibodies raised in rabbits immunized with murine PLA2R induced the histological features of MN and proteinuria. The transfer of patient autoantibodies by contrast did not induce these changes. This can be attributed to the fact that the human anti-PLA2R antibodies did not efficiently bind the murine protein, which shares an amino acid homology of only 72% with human PLA2R. Interestingly, patient-derived autoantibodies activated the complement system and induced cytotoxicity in an in vitro cell system (Lateb et al. 2019). Together, the formal in vivo proof of the pathogenicity of human anti-PLA2R antibodies in MN is still missing.

### Perspectives in MN pathomechanisms

Several studies have convincingly shown that antibodies to podocyte membrane antigens can cause MN in animals after in situ binding to a podocyte resident antigen (Heymann 1952; Debiec et al. 2002; Tomas et al. 2016, 2017; Meyer-Schwesinger et al. 2020). However, the molecular mechanisms that mediate podocyte damage after binding of the antibodies to the target antigen on podocytes are incompletely understood. In particular, the role of complement versus complement-independent mechanisms needs further experimental workup in the future.

#### Role of complement

In the Heymann nephritis models, binding of antibodies to their target antigen leads to the activation of the complement cascade with formation of the final product C5b-9, also known as the membrane attack complex. C5b-9 is inserted into podocyte membranes, resulting in a sublethal cell damage (Kerjaschki 1992). In the passive Heymann nephritis model, depletion of complement C3 by cobra venom factor and depletion of complement C6 by anti-C6 antibodies prevented the development of proteinuria, suggesting an essential role of the complement system in the mediation of proteinuria (Salant et al. 1980; Baker et al. 1989). However, other studies found no impact on proteinuria when complement was removed, challenging this pathophysiological concept (Leenaerts et al. 1995; Spicer et al. 2007). Additionally, the lack of detectable complement deposition in the anti-THSD7A antibody transfer models, at least during the heterologous phase of disease, hint to the existence of complement-independent injury mechanisms in MN. Worthy of note, these models face several limitations: First, the classic protocols for both active and passive Heymann nephritis involve antibodies against a multitude of tubular and glomerular proteins, limiting the antigen-specificity of the findings. Second, the applied passive models involve the transfer of foreign antibodies with potentially limited complement-activating capacity. Third, the transfer of the heterologous antibodies causes a rapid onset of disease, which does not reflect the situation in patients with MN, where disease is evolving slowly, likely over the course of several months or even years (Burbelo et al. 2020). The establishment of experimental MN models involving active immunization with a single podocyte antigen (i.e., PLA2R or THSD7A) represents a strategy to overcome the issues of lacking antigen specificity, complement activating capacity of foreign IgG and hyperacute dynamics of disease onset, which will be essential to further dissect the role of complement in the pathogenesis of MN.

Until now, it is unclear via which pathway the complement system is activated in patients with MN. In principal, the complement system can be activated via the classical, the lectin and the alternative pathway. All three pathways converge into C3 with a common final pathway, leading to the formation of C5b-9 (Fig. 3, right). Typically, autoantibody binding to an antigen activates the classical pathway by binding of C1q. However, in MN this assumption is challenged by the situation that IgG4 is the dominant autoantibody subclass in patients with PLA2R- and THSD7A-associated MN. IgG4 is the IgG subclass that shows the least binding to C1q and thus has limited (or no) capacity to activate the complement system via the classical pathway (Vidarsson et al. 2014). In line with this fact, C1q was described to be absent in cases of primary MN (Jennette et al. 1985), a finding which is contradicted by other studies (Wiech et al. 2019). The presence of C4d in the absence of C1q may hint to a role of the lectin pathway (Val-Bernal et al. 2011). The lectin pathway can be initiated by binding of mannan-binding lectin to a hypogalactosylated IgG with subsequent cleavage of C4 (Malhotra et al. 1995). Recently, differentially glycosylated anti-PLA2R antibodies of the IgG4 subclass have been demonstrated to bind MBL in vitro, suggesting that the lectin pathway may be directly activated by anti-PLA2R IgG4 (Haddad et al., 2020). However, MN can also occur in patients with a genetic MBL deficiency, suggesting involvement of pathways other than the lectin pathway (Bally et al. 2016). Noteworthy, PLA2R- and THSD7A-specific IgG subclasses other than IgG4 are also present in patient biopsies and sera, which could account for glomerular complement activation (Huang et al. 2013; von Haxthausen et al. 2018). A recent study applied mass spectrometry to detect complement components as well as complement regulatory proteins in patients with PLA2R- and EXT1/EXT2-associated MN (Ravindran et al. 2020). C3 was found to be the most abundant complement component and C4, C5, C6, C7, C8, and C9 were also present, while complement components upstream of C3, which could help to understand complement initiation in MN, were less abundant. Thus, complementary methods are needed to dissect the specific role of the complement activation pathways for disease initiation and prognosis of patients with MN.

#### Role of complement-independent mechanisms

NEP is a cell-surface expressed metalloprotease that is involved in the inactivation of several peptide hormones (Turner et al. 2001). It could be demonstrated that anti-NEP IgG1 from alloimmunized mothers strongly inhibited NEP enzymatic function additionally to the activation of complement and the diseased children had a more severe disease (Vivarelli et al. 2015). Thus, interference of MN autoantibodies with the enzymatic activity of the antigen constitutes a potential complement-independent pathogenic mechanism in MN cases.

The precise function of PLA2R, in particular in the podocyte, is largely unknown. It was shown that PLA2R serves as a receptor for secretory phospholipases (sPLAs), which are pro-inflammatory enzymes. Thereby, PLA2R might act in two directions, as a clearance receptor or as a positive regulator of sPLA2. The interaction of PLA2R with sPLAs can lead to different effects, for example protein kinase activation, producing lipid activators or activating DNA damage pathways (Lambeau et al. 2008; Augert et al. 2009). Additionally, PLA2R was identified to interact with the annexin 2/S100A10 complex, which is involved in organization of the actin cytoskeleton (Fresquet et al. 2017). Anti-PLA2R antibodies may interfere with such functions, contributing to glomerular damage in MN. Furthermore, PLA2R was proposed to alter the migratory responses to collagen types I and IV. Recombinant PLA2R binds to collagen I and thereby interrupts the interaction between collagen and integrin beta 1, thus decreasing beta 1 integrin–mediated migration (Takahashi et al. 2015; Watanabe et al. 2018a, b). One could speculate that covering of PLA2R with antibodies may interrupt the interaction of PLA2R with collagen I, which in turn may enhance collagen I binding to beta 1 integrin and thus integrin beta 1–mediated migratory activity with the consequence of destabilization of the GFB. However, the occurrence of such mechanisms and a potential pathophysiologic role in patients with MN remain to be experimentally proven.

The biological role of THSD7A is also poorly understood. Earlier studies demonstrated that THSD7A mediates cell migration and tube formation during angiogenesis in association with focal adhesion proteins in endothelial cells (Wang et al. 2010; Kuo et al. 2011). Recently, it was shown that THSD7A expression is pronounced at the basal aspect of podocyte foot processes and that THSD7A overexpression in cell culture podocytes is associated with increased cell size and cell adhesion to collagen IV as well as a decreased cell migratory potential (Herwig et al. 2019). Thus, disruption of cell adhesion through binding of anti-THSD7A antibodies to THSD7A represents another interesting potential disease mechanism.

Both PLA2R and THSD7A are large multidomain membrane proteins expressed on podocyte foot processes, likely interacting with other surrounding molecules. For example, PLA2R was shown to interact with collagen type IV (the collagen subtype that is the main constituent of the GBM) (Takahashi et al. 2015; Watanabe et al. 2018a, b), and the TSP-1 domains of THSD7A may interact with heparan sulfate (an essential component of the GBM) (Stoddard et al. 2019). Thus, it is possible that PLA2R and THSD7A have essential structural functions, contributing to the integrity of the GFB by anchoring podocyte foot processes to the outer aspect of the GBM. Anti-PLA2R (Skoberne et al. 2014) and anti-THSD7A antibodies might directly interfere with such a structural function. In the heterologous phase of the THSD7A-associated mouse model of MN, it will be possible to investigate this hypothesis directly, e.g. in high-resolution immunofluorescence imaging and ultrastructural electron microscopy analyses of the GFB. However, as PLA2R seems to be dispensable for GFB integrity in mice, such investigations are limited to THSD7A.

Both the receptor function of PLA2R and the association of THSD7A with focal adhesions suggest that these molecules are involved in specific signaling programs, potentially through their short cytoplasmic tails, and it seems possible that antibody binding interferes with such signaling. However, no such signaling pathways have been identified to date and the heterologous models of PLA2R- and THSD7A-associated MN may be applied to elucidate such potential pathomechanisms. After the disease is induced by transfer of rabbit antibodies, whole glomeruli or even podocytes, endothelial cells and mesangial cells can be isolated using FACS-sorting (Boerries et al. 2013). The isolated samples can be investigated for signaling events, e.g., by performing transcriptomic and proteomic analyses. This may reveal PLA2R- and THSD7A-specific signaling pathways that are activated or deactivated by the respective antibodies. The identified targets can be further investigated regarding their pathogenic relevance, e.g., by generating respective knockout mice. Finally, such pathways may serve as therapy targets, which can be evaluated in the models of PLA2R- and THSD7A-associated MN.

## Clinical use of autoantibody measurement to guide diagnosis and therapy

Since the identification of PLA2R and THSD7A as target antigens in MN, diagnosis and treatment of patients has been adapted to the pathophysiology of disease (De Vriese et al. 2017). Staining of renal tissue for both target antigens as well as the detection of the corresponding autoantibodies in the blood have become standard techniques. For the detection of anti-PLA2R and anti-THSD7A antibodies, a number of tests have been developed, i.e., Western blot, indirect immunofluorescence tests, ELISAs, or a chemiluminescence immunoassay, some of which are widely used in clinical routine (Beck et al. 2009; Hoxha et al. 2011, 2017; Hofstra et al. 2012; Dahnrich et al. 2013; Tomas et al. 2014; Seitz-Polski et al. 2018; Zaghrini et al. 2019). The detection of antibodies in the serum is very specific for the diagnosis of MN, even leading to a discussion whether a renal biopsy is still required in the presence of detectable anti-PLA2R or anti-THSD7A antibodies in nephrotic patients (De Vriese et al. 2017; Bobart et al. 2019; Wiech et al. 2019).

Both PLA2R and THSD7A show a characteristic granular positivity in kidney biopsies of patients with PLA2R and THSD7A-associated MN, respectively (Hoxha et al. 2012; Tomas et al. 2014). Both antigens colocalize with IgG in the immune deposits and allow a correct diagnosis of the disease in virtually 100% of cases, even if antibodies are not detectable in the blood, i.e. in patients after spontaneous or immunosuppressive-induced remission, or in cases when proteinuria develops before the antibodies become detectable in the blood (Beck et al. 2009; Svobodova et al. 2013). Although the presence of the autoantibodies confirms the diagnosis of MN, a renal biopsy is still routinely performed in most patients with no contraindication to the procedure, since it allows an estimation of the individual prognosis and diagnosis of concurrent diseases (Wiech et al. 2019). Therefore, taking into account individual factors has become more relevant when making the decision whether or not to perform a kidney biopsy in nephrotic patients with detectable anti-PLA2R antibodies. Such factors include, for example, whether a concurring renal disease is likely based on medical history and urinalysis, whether renal function is impaired, or the individual risks of the procedure (bleeding, use of anticoagulants, etc.).

Monitoring of anti-PLA2R antibodies is widely used to guide treatment decisions. Higher antibody levels are associated with a higher risk for loss of renal function, recurrence of disease after renal transplantation, relapse of proteinuria and a lower chance to develop a remission of disease both spontaneously or on immunosuppressive treatment (Kanigicherla et al. 2013; Bech et al. 2014; Hoxha et al. 2014a, c; Dahan et al. 2018; Xipell et al. 2018; Mahmud et al. 2019). Therefore, the previous management of MN patients with an initial waiting period of 6 months prior to starting an immunosuppressive treatment is shifting to a more pathophysiology-driven treatment approach. Since patients with very high anti-PLA2R antibody levels, impaired renal function and high level proteinuria have the worst prognosis, an early treatment start might be beneficial. Currently, a “preemptive” immunosuppressive treatment in patients with anti-PLA2R antibodies but low proteinuria (proteinuria < 3.5 g/day) is usually not recommended, although some cohort studies have shown that such patients often develop nephrotic-range proteinuria (Hoxha et al. 2014b). Considering the time lag between development of antibody titers and the onset of proteinuria, a close monitoring of these patients is needed in order to differentiate whether patients will develop a remission of antibodies or a relapse of proteinuria. A spontaneous remission of proteinuria is especially common in patients with a median anti-PLA2R antibody level of 50 U/ml, while patients with a median anti-PLA2R antibody level of 150 U/ml usually need an immunosuppressive treatment (Mahmud et al. 2019). Such individualized patient management approaches have become possible because of the close association of anti-PLA2R antibody levels with disease activity and outcome – a circumstance that represents additional evidence (albeit no proof) that anti-PLA2R antibodies are pathogenically relevant. Immunosuppressive treatment of MN patients is still mainly based on three agents: alkylating drugs, calcineurin inhibitors, and B cell depleting agents. In all cases, different treatment protocols exist and there is no agreement whether one treatment (protocol) is particularly suitable for patients with PLA2R-associated MN. This is mainly because all MN studies performed until now have failed to take into account the anti-PLA2R antibody status or level, neither in their recruiting, nor stratification strategy. Additionally, there is no study that has investigated the adjustment of immunosuppressive dosage or treatment termination based on the anti-PLA2R antibody level. Such an approach has the potential to significantly reduce treatment intensity and, therefore, treatment toxicity, and improve the prognosis of MN. Glomerular immune deposits remain detectable for many months, or even years, after anti-PLA2R antibodies have become negative in the circulation. Therefore, proteinuria may persist for a period of time in these patients, even though the immunological remission has already been achieved. In this context, proteinuria is not a reliable clinical parameter, and termination of immunosuppressive treatment when anti-PLA2R antibodies become undetectable in the blood is a pathophysiologically reasonable approach. To enable such a strategy, the development and application of detection systems with a high sensitivity for anti-PLA2R antibodies are necessary. On the other hand, in patients with no depletion of anti-PLA2R antibodies after immunosuppressive therapy, proteinuria usually persists or relapses (Bech et al. 2014). Therefore, a maintenance immunosuppressive therapy might be warranted in these patients. Clinical studies investigating the outcome of such patients as well as the best maintenance immunosuppressive therapy are still lacking. Such studies may help to further personalize treatment decisions in patients with MN, e.g., whether treatment should be continued until anti-PLA2R antibodies are undetectable, which immunosuppressant is best suited for maintenance therapy, etc.

Anti-PLA2R and anti-THSD7A antibodies target multiple epitopes within different extracellular domains of the respective molecules (Fresquet et al. 2015; Seitz-Polski et al. 2016; Seifert et al. 2018; Reinhard et al. 2020). It is still controversial whether the different PLA2R epitope recognition patterns of autoantibodies play a prognostic role or influence treatment response in patients with PLA2R-associated MN (Seitz-Polski et al. 2018; Reinhard et al. 2020). The different findings in these manuscripts may result from the different techniques used to detect domain-specific antibodies. The total anti-PLA2R antibody level was shown in a number of studies to be the best biomarker for prognosis of outcome in patients with MN (Kanigicherla et al. 2013; Ruggenenti et al. 2015; Dahan et al. 2017; Mahmud et al. 2019). The clinical role of domain-specific anti-THSD7A antibodies has not been adequately evaluated yet, mainly due to the low incidence of THSD7A-associated MN (Seifert et al. 2018).

The antigen-driven diagnosis of MN has also improved the differential diagnosis and screening procedures performed in these patients. The description of patients with THSD7A-associated MN and concomitant THSD7A-expressing tumors, combined with the finding that patients with THSD7A-associated MN show a higher tumor prevalence, has led to the recommendation to more thoroughly screen patients with THSD7A-associated MN for the presence of tumors (Hoxha et al. 2016, 2017; Lin et al. 2018; Matsumoto et al. 2019; Taguchi et al. 2019; Wang et al. 2019; Weinmann-Menke et al. 2019; Xian et al. 2019; Zhang et al. 2019).

## Innovative strategies to treat MN

Alkylating agents combined with steroids are an effective treatment for patients with MN and have been the mainstay of treatment for decades (Ponticelli et al. 1984). Cyclosporine A has also been widely used as an alternative regimen, but this treatment is associated with an increased rate of disease relapse (Cattran et al. 1995, 2001). Both treatments involve broad immunosuppression leading to a decrease in autoantibody levels, but also contain substantial potential adverse effects, in particular in the case of alkylating agents. Given the antibody-mediated pathogenesis of MN and the role of anti-PLA2R antibody measurement to monitor disease activity and response to treatment, the use of more targeted therapeutic approaches becomes obvious. Some groups have applied plasmapheresis to eliminate the pathogenic antibodies (Muller-Deile et al. 2015; Weinmann-Menke et al. 2019; Podesta et al. 2020), yet this strategy was only successful in few patients, which usually additionally received immunosuppressive treatment to suppress antibody production. The exact role of plasmapheresis in the treatment of (refractory) cases of MN has still to be defined. Since its first use for treatment of MN in 2002 (Remuzzi et al. 2002), the B cell-depleting monoclonal antibody rituximab was shown to be effective in MN patients. Interestingly, rituximab is associated with fewer adverse events than the combination of steroids with cyclophosphamid (van den Brand et al. 2017). Recently, the first randomized controlled trials have shown treatment efficacy and safety (Dahan et al. 2017; Fervenza et al. 2019). In the MENTOR trial, rituximab was shown to be superior to cyclosporine after 24 months (Fervenza et al. 2019). The ongoing RI-CYCLO trial compares the use of rituximab with cyclophosphamide for patients with MN, but so far only 76 patients have been included (www.clinicaltrials.gov). Moreover, higher dosages of rituximab may be needed, especially in highly proteinuric patients for treatment to be effective, since rituximab may be lost in the urine. Studies investigating the best dosage regimen for rituximab and taking into consideration proteinuria and anti-PLA2R antibody levels are lacking. However, a retrospective study showed an association of higher rituximab dosage (i.e., 2 × 1 g of rituximab versus 2 × 375 mg/m^2^ body surface area) with earlier and increased remission rates in patients with MN and suggested that monitoring of the serum rituximab levels may be a way to guide effective treatment (Boyer-Suavet et al. 2019; Seitz-Polski et al. 2019).

Additionally, rituximab is not an antigen-specific treatment as it depletes all B cells with potential unfavorable consequences such as hypogammaglobulinemia and infections.

The molecular characterization of the major MN antigens and the better understanding of the role of autoantibodies for MN pathophysiology may lead to antigen-specific treatment strategies of MN.

### Perspectives in MN treatment

One strategy is the inhibition of the complement system. This treatment strategy is particularly interesting since it would interrupt ongoing complement-mediated glomerular damage at the end of the injury cascade, potentially preventing further glomerular damage or allowing more efficient glomerular recovery. Such treatments may also apply as an add-on strategy to the removal of the pathogenic antibodies. However, blockade of the terminal complement cascade (e.g., with the anti-C5 monoclonal antibody eculizumab) could interfere with the effect of rituximab, since one of the mechanisms of action of rituximab to eliminate B cells relies on complement-dependent cytotoxicity (Reff et al. 1994). In addition, the pathogenic role of complement, especially the role of the different complement activation pathways, has not finally been defined in MN, and such a treatment approach does not account for potential complement-independent pathogenic mechanisms in MN. A preliminary trial of eculizumab in patients with MN was not successful. Recently, LNP023, an inhibitor of the alternative pathway, was shown to reduce proteinuria in the passive Heymann nephritis model (Schubart et al. 2019). This is interesting as the alternative pathway is often considered an amplification loop of the classical pathway (Harboe et al. 2004). Whether the inhibition of the alternative pathway (amplification loop) will be sufficient to achieve a substantial and lasting therapeutic effect in patients with MN if autoantibodies persist in the circulation will have to be studied in prospective studies. A clinical trial comparing the efficacy of LNP023 to rituximab is currently recruiting patients (www.clinicaltrials.gov) and the results will surely be awaited eagerly by the field.

**Fig. 4:**
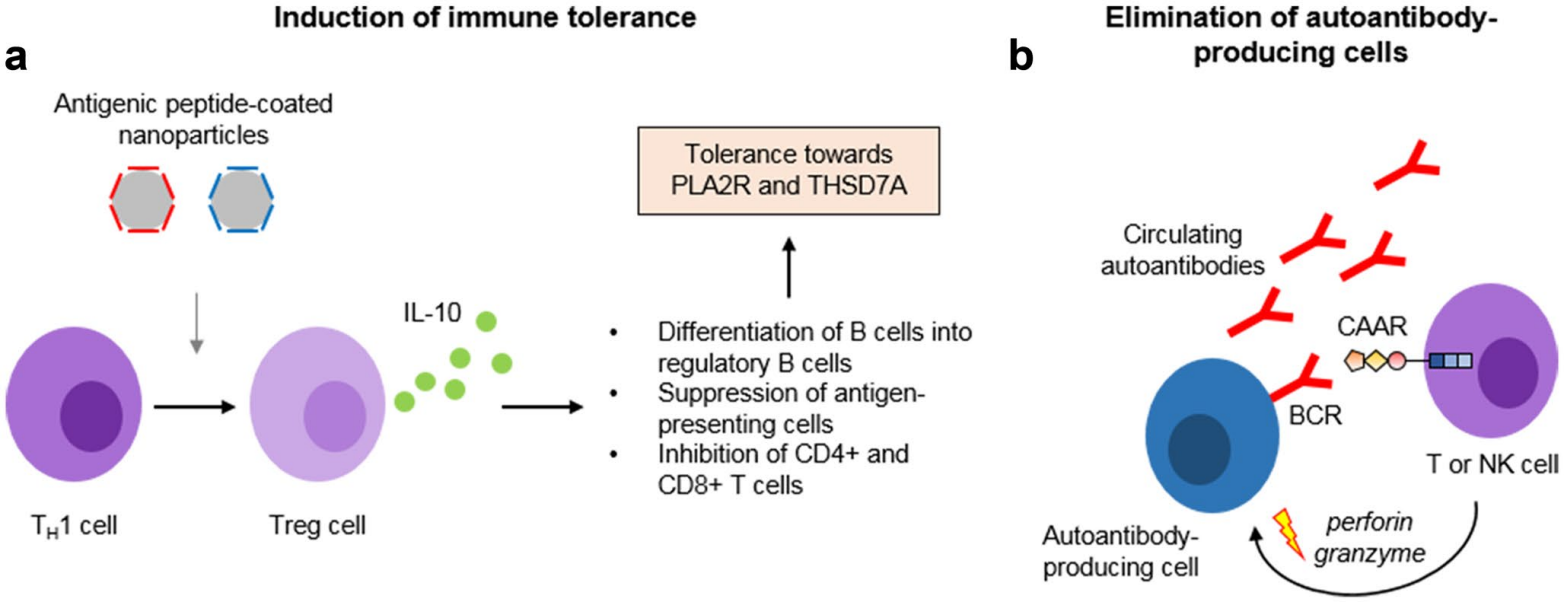
Innovative strategies to treat MN. (**a**) Antigen-specific tolerance can be induced using nanoparticles coated with antigen peptides corresponding to the T cell epitopes. Such nanoparticles can induce the differentiation of proinflammatory TH1 cells to regulatory T (Treg) cells, which produce interleukin-10 (IL-10). IL-10 induces the differentiation of B cells to regulatory B cells, the suppression of antigen-presenting cells and the inhibition of different T cell populations, collectively decreasing the immune response against the respective specific antigen. (**b**) Autoantibody-producing cells can be eliminated based on the specificity of the B cell receptor (BCR). The BCR is a membrane-bound antibody, corresponding to the antibody that is produced by this exact cell. T and NK cells can be transduced with chimeric autoantibody receptors, consisting of antigen fragments coupled to a transmembrane and several intracellular signaling domains. Depicted is a CAAR containing the first three domains of PLA2R, the cysteine-rich domain, the fibronectin type II domain, and the first c-type lectin domain. Binding of the CAAR-transduced cell to the BCR induces signaling events leading to the release of granzyme and perforins, which can efficiently eliminate the nearby autoantibody-producing cell

A second strategy is the regaining of immune tolerance through induction of Tregs. This has successfully been performed using nanoparticles that are coated with antigenic peptides (Clemente-Casares et al. 2016). These nanoparticles induce antigen-specific Tregs, which in turn promote the differentiation of B cells into regulatory B cells, efficiently suppress antigen-presenting cells, and inhibit CD4+ and CD8+ T cells, collectively leading to the resolution of autoimmune disease (Fig. 4a). In a similar approach, the microparticles can be directed to the liver, where they are taken up by liver sinusoidal epithelial cells (LSECs), leading to expansion of Tregs and strong amelioration of autoimmune disease, in this case experimental autoimmune encephalomyelitis (Carambia et al. 2015).

One promising therapeutic strategy to eliminate pathogenic B cell clones is the generation of chimeric autoantibody receptor (CAAR) T or NK cells, a modification of the oncotherapeutic strategy of chimeric antigen receptor (CAR) T cells (June et al. 2018). CAR T cell therapy involves the isolation of peripheral blood mononuclear cells (PBMCs) from a patient’s blood using leukapheresis. Stimulation of T cells using interleukin-2 and anti-CD3 antibodies leads to their proliferation. The T cells are then transduced with a construct encoding for the CAR of interest, e.g., the antigen-binding domain of an anti-CD19 antibody fused to intracellular signaling domains (June et al. 2018). The resulting CAR T cells are transfused back to the patient, serving as a “living drug” to eliminate all cells expressing, in this case, CD19, a marker of B cells. This therapeutic strategy has been used with remarkable success, for example in refractory or relapsed B cell lymphoma (Maude et al. 2018; Park et al. 2018; Schuster et al. 2019). For treatment of antibody-mediated autoimmune diseases, this approach can be modified by expressing a CAAR on T or NK cells. In contrast to a CAR, which recognizes a certain surface antigen, the CAAR contains domains of the antigen of interest (e.g., autoantibody-binding domains of PLA2R or THSD7A), enabling it to bind the corresponding antigen-specific B cell receptor (Fig. 4b). Thus, the engineered CAAR T or NK cell will bind to the B cell of interest, leading to its elimination. In an animal model of pemphigus vulgaris, CAAR T cells specifically and efficiently eliminated anti-desmoglein 3-specific autoreactive B cells even in the presence of circulating autoantibodies and without relevant off-target toxicity (Ellebrecht et al. 2016). However, the circulating autoantibodies may be an obstacle that has to be overcome for efficient CAAR T or NK cell action as the autoantibodies, by binding to the antigen part of the CAAR, may inactivate or even eliminate the injected CAAR T or NK cells. This could be achieved by immunoadsorption of the specific autoantibodies (Hamilton et al. 2018), resulting in a time window of optimized CAAR T or NK cell action.

## Conclusions

Over the past 10 years, substantial novel insights into the pathophysiology of MN have been gained. In particular, the identification of target antigens and their extensive clinical and pathophysiological characterization has led to a novel molecular perspective on this disease. Further implementation of this knowledge together with an even deeper understanding of the underlying pathomechanisms may lead to a paradigm shift towards a more targeted and less toxic treatment.
